# Efficacy and Safety of Different Local Anesthesia Techniques in Podiatric Procedures

**DOI:** 10.3390/medicina61040588

**Published:** 2025-03-25

**Authors:** Bibiana Trevissón Redondo, Nathael Lago García, Rubén García Fernández, Hector Pereiro Buceta, Roi Painceira Villar, Alberto Gonzalez Garcia, David Bermejo Martínez, Natalia Calvo-Ayuso, Enedina Quiroga-Sánchez

**Affiliations:** 1 SALBIS Research Group, Faculty of Health Sciences, Department of Nursing and Physiotherapy, Campus de Ponferrada, Universidad de León, 24401 León, Spain; btrer@unileon.es (B.T.R.); ncala@unileon.es (N.C.-A.); equis@unileon.es (E.Q.-S.); 2 PhD School, Universidad de León, 24401 León, Spain; 3 Nursing Research, Innovation and Development Centre of Lisbon (CIDNUR), Nursing School of Lisbon, 1600-190 Lisbon, Portugal; 4 Department of Nursing and Physiotherapy, Campus de Ponferrada, Universidad de León, 24401 León, Spain; hperb@unileon.es (H.P.B.); rpaiv@unileon.es (R.P.V.); dbermm@unileon.es (D.B.M.); 5 Department of Nursing and Physiotherapy, Campus de Vegazana, Universidad de León, 24071 León, Spain; agong@unileon.es

**Keywords:** anesthesia, pain control, podiatry, patient satisfaction, safety

## Abstract

*Background and Objectives:* Local anesthesia is essential in podiatry, ensuring painless procedures. Technological and pharmacological advances require us to stay updated on the safest and most effective techniques. Lidocaine and bupivacaine are common anesthetics in this field, with the choice of technique tailored to each procedure. To evaluate the effectiveness and safety of local anesthesia techniques in reducing pain in podiatric procedures. *Materials and Methods*: A systematic review followed PRISMA guidelines, searching for studies in PubMed, Scopus, and Web of Science published in the last 10 years in English and Spanish. Studies focused on local anesthesia in podiatry were included, excluding those unrelated or without validated results. *Results:* Of 485 initial studies, nine were selected that met all criteria. These studies demonstrated the efficacy and safety of various local anesthesia techniques, such as WALANT and ultrasound-guided peripheral nerve blocks, highlighting their effectiveness in pain control and patient satisfaction. *Conclusions:* Local anesthesia techniques are effective in reducing pain in podiatric procedures. The safety of these techniques is high, with few serious complications. Local anesthesia without a tourniquet and specific techniques, such as subparaneural injection, are effective for pain control. Individual patient factors and surgeon experience influence results.

## 1. Introduction

Local anesthesia represents a cornerstone in podiatric practice, ensuring pain-free interventions and significantly enhancing the quality of patient care [1]. The selection of appropriate techniques and anesthetic agents is crucial for the success of any podiatric procedure, ranging from callus removal to more complex surgical interventions [2,3]. In a field where technological and pharmacological advancements evolve rapidly, it is imperative to stay informed about the most effective and safest methodologies to ensure optimal patient outcomes.

Among the anesthetics commonly used in podiatry are lidocaine and bupivacaine, both recognized for their efficacy in blocking nerve transmission, thereby allowing painless procedures [2,4]. Due to its rapid onset of action (approximately 5 to 10 min after application) and an effective duration of 1 to 2 h, Lidocaine is ideal for short procedures, such as plantar wart removal or ingrown toenail treatment [1,2]. On the other hand, bupivacaine, which has a slower onset but a prolonged duration of 4 to 8 h, is preferred for more complex surgical interventions, such as foot deformity correction or foreign body extraction [4].

It is essential to consider the concentration of these agents, as variations can significantly influence both the duration and depth of anesthesia. Additionally, the combined use of a vasoconstrictor, such as epinephrine, can prolong the anesthetic effect and reduce local bleeding, particularly useful in incision procedures [5].

The choice of anesthetic technique should also be adapted to the type of procedure and the specific area to be treated [4,6]. Techniques such as infiltrative anesthesia, where the anesthetic is injected directly into the soft tissue surrounding the treatment area, and regional anesthesia, which blocks nerves in a larger area, are commonly used in podiatry [7]. The former is frequently utilized to remove small skin lesions or biopsies, while the latter is more appropriate for extensive foot and ankle surgeries [3].

The appropriate selection of anesthesia in podiatric procedures is decisive in ensuring patient comfort and optimizing the surgical intervention’s effectiveness. According to Benson and Benson, preoperative considerations for podiatric surgical candidates should encompass the patient’s overall physical condition and specific anesthesia-related factors that may influence the selection of the most appropriate technique [1]. Skully et al. emphasize the importance of a comprehensive preoperative evaluation that incorporates current trends in patient management, which is essential for identifying potential risks and ensuring a personalized approach to anesthesia administration [2].

Reilley and Gerhardt highlight the diversity of anesthetic techniques applicable in foot and ankle surgeries, stressing the need to tailor the anesthetic approach to the specific characteristics of each case [3]. This point is supported by studies such as that conducted by Wang et al., which examines pain management in elective foot and ankle surgeries, suggesting the efficacy of evidence-based approaches in improving postoperative outcomes [4].

As Kohring and Orgain described, multimodal analgesia emerges as a prominent strategy for effective pain management in foot and ankle surgeries, emphasizing the importance of an integrative approach that combines different pain control methods [5]. MacNeill and Mayich provide evidence on the efficacy and safety of foot and ankle surgery performed under local anesthesia, highlighting the feasibility of less invasive techniques for optimizing postoperative recovery [6].

It is also essential to mention the research conducted by Robertson et al., which explores the impact of prolonged tourniquet times on wound healing in foot surgeries. This research points to the relevance of selecting anesthetic techniques that minimize potential postoperative complications [7]. Furthermore, as detailed by Stéfani et al., postoperative analgesia using peripheral nerve blocks constitutes an effective method for pain relief, ensuring a more comfortable recovery for the patient [8].

Finally, preoperative pain management and the prevention of chronic postoperative pain, as investigated by Stiegelmar et al. (2019), along with the comparative analysis by Dang et al. (2019) between single-agent regional anesthesia and regional anesthesia with three additives, underscore the continuous evolution of anesthetic practices in podiatry aimed at improving patient care quality and long-term outcomes [9,10].

Considering that the appropriate selection of anesthesia and its application technique are fundamental in ensuring patient comfort and the success of podiatric interventions, this review aims to assess the efficacy and safety of various local anesthetic techniques used in podiatric procedures.

However, despite the widespread use of local anesthesia in podiatric procedures, no standardized guideline clearly defines which techniques should be applied for each type of intervention. The wide variety of anesthetic techniques, the diversity of agents used, and the differences in how these techniques are implemented based on surgeon experience contribute to significant heterogeneity in clinical practice. This lack of consensus creates uncertainty for practitioners and may affect patient outcomes. Thus, this systematic review seeks to fill this gap by identifying, comparing, and evaluating the efficacy and safety of different local anesthesia techniques in podiatry to provide evidence-based guidance that supports clinical decision making and promotes standardized, high-quality care. This specialized knowledge ensures that podiatrists can perform a wide range of procedures effectively and safely, adapting to each patient’s needs.

## 2. Materials and Methods

A systematic and rigorous methodological approach was adopted to conduct this comprehensive review on the efficacy and safety of local anesthesia techniques in podiatric procedures. The PRISMA guidelines (Preferred Reporting Items for Systematic Reviews and Meta-Analyses) [11] ensured transparency and consistency in reporting and analyzing the collected data. This framework is widely recognized and accepted in the scientific community for its ability to enhance the quality of systematic reviews and meta-analyses. In addition, to increase transparency, avoid duplication, and improve the quality and visibility of the research, it was registered in PROSPERO under the registration number CRD42025640577.

A comprehensive literature search was conducted in several recognized electronic databases, including PubMed, Scopus, and Web of Science, covering sources in both English and Spanish with a temporal scope of the last 10 years to ensure the inclusion of the most relevant and up-to-date information. The selected search terms, derived from the MeSH thesaurus, were combined using the Boolean operators AND and OR. The resulting search equations were aligned with the following specific research objectives:

To evaluate the efficacy of different local anesthesia techniques in reducing pain during podiatric procedures: (“Anesthesia, Local” (Mesh) OR “Local Anesthetics” (Mesh)) AND (“Pain, Postoperative” (Mesh) OR “Pain Measurement” (Mesh) OR “Pain Management” (Mesh) OR “Pain Threshold” (Mesh)).

To analyze the safety of different local anesthesia techniques used in podiatry: (“Anesthesia, Local” (Mesh) OR “Local Anesthetics” (Mesh)) AND (“Adverse Effects” (Mesh) OR “Drug-Related Side Effects and Adverse Reactions” (Mesh) OR “Drug Toxicity” (Mesh) OR “Anesthesia, Conduction” (Mesh) OR “Anesthesia, Epidural” (Mesh)).

To compare the relative efficacy and safety of different types of local anesthetics, administration techniques, and dosages used in podiatric procedures: (“Anesthesia, Local” (Mesh) OR “Local Anesthetics” (Mesh)) AND (“Drug Administration Routes” (Mesh) OR “Drug Dosage Calculations” (Mesh) OR “Drug Dosage Forms” (Mesh) OR “Drug Dosage Time Course” (Mesh) OR “Anesthesia, Local/adverse effects” (Mesh) OR “Anesthesia, Local/methods” (Mesh)).

To identify potential factors that may influence the efficacy and safety of local anesthesia techniques in podiatry: (“Anesthesia, Local” (Mesh) OR “Local Anesthetics” (Mesh)) AND (“Risk Factors” (Mesh) OR “Prognosis” (Mesh) OR “Patient Selection” (Mesh) OR “Practice Guidelines as Topic” (Mesh) OR “Professional Practice” (Mesh) OR “Treatment Outcome” (Mesh)).

Clear and justified inclusion and exclusion criteria were established to ensure the integrity and applicability of the review results. The inclusion criteria encompassed studies that focused exclusively on the efficacy and safety of local anesthesia techniques used in podiatric procedures, were published within the last 10 years to ensure data relevance and currency, were available in English or Spanish to facilitate detailed analysis and interpretation, and included any study design, from randomized clinical trials to observational studies, to obtain a comprehensive perspective on the topic.

Conversely, studies were excluded if they did not provide specific data on the efficacy or safety of local anesthesia techniques in podiatry, presented unconfirmed or preliminary results that had not been validated in subsequent studies, were narrative reviews, expert opinions, or editorials lacking direct empirical evidence, or addressed anesthesia techniques not applicable to podiatric procedures or focused on populations not directly related to podiatry.

To ensure transparency and precision in the selection of relevant studies, the screening and selection process was initially conducted by a primary researcher. This process involved an initial review of titles and abstracts to identify potentially eligible studies based on predefined inclusion criteria. Those meeting these initial requirements underwent a full-text review to confirm their eligibility and relevance.

Although a single researcher performed the initial screening, two additional reviewers supervised and validated the process. Their oversight helped to minimize potential bias and ensured that the inclusion and exclusion criteria were applied consistently. All decisions were meticulously documented using the Zotero tool, and reasons for exclusion were explicitly justified for non-selected studies. This collaborative approach strengthened the objectivity and reliability of the literature selection process, essential for compiling robust evidence to support the research objectives.

## 3. Results

During the search process, 485 bibliographic references were identified and downloaded into Zotero to verify inclusion and exclusion criteria. The retrieved records were initially sorted, and 59 duplicates were excluded, reducing the dataset to 426.

The records were then arranged by date, confirming that all had been published after 2014, meeting the inclusion requirement. Subsequently, the language of each study was checked by reviewing the title, and no studies were preliminarily excluded based on this criterion.

The screening process continued with a review of the study titles to ensure their alignment with the objectives of this review, specifically investigating the effectiveness and safety of local anesthesia in podiatric procedures. 128 studies were excluded as they addressed anesthesia but did not specifically focus on podiatry. In comparison, 74 studies were eliminated for being related to podiatry but not centering on anesthesia.

The abstracts of the remaining 224 studies were reviewed to assess their relevance to this study. 65 studies were excluded from being reviewed, and 23 were removed due to their qualitative focus. Additionally, 83 studies were excluded for not specifying the methodology used or failing to employ validated tools for presenting their results.

**Figure 1:**
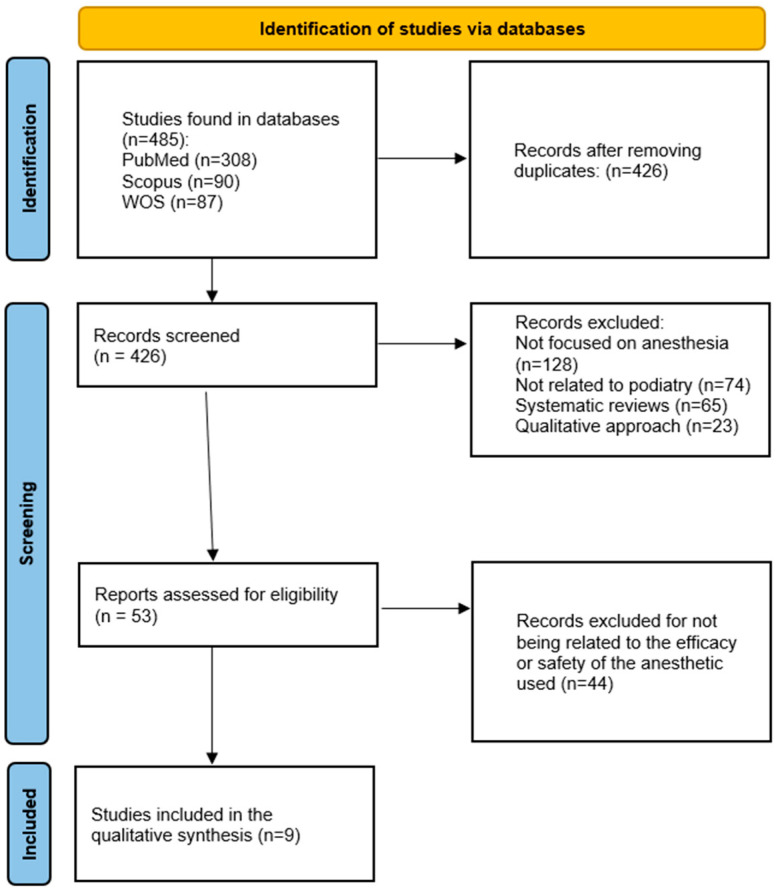
Flowchart of the selection process.

The remaining 53 studies underwent full-text review. Nine studies were ultimately selected for qualitative synthesis. Figure 1 summarizes the screening process implemented for this review, and Table 1 summarizes the results.

The study conducted by Bilgetekin et al. [12] retrospectively analyzed the effectiveness of the WALANT (Wide-Awake Local Anesthesia No Tourniquet) technique in 31 patients with foot and ankle injuries, including 22 men and nine women with a mean age of 40 ± 16 years. The treated injuries included 15 medial malleolus fractures, five lateral malleolus fractures, five Achilles tendon ruptures, two proximal phalanx fractures, and less common ones, such as Lisfranc and fifth metatarsal fractures. Of the patients, 27 were classified as ASA I-II and four as ASA III. The mean operation duration was 36.6 ± 7 min, and the average hospital stay was 8.3 ± 6.1 h. Patients reported a median pain score of 1 (range 0–4) and anxiety score of 1 (range 0–3) on the VAS scale, with no need for additional anesthetics or intensive care and no postoperative complications. These results suggest that WALANT is an effective and safe technique, providing adequate pain and anxiety management along with rapid hospital recovery.

Çetin et al. [13] conducted a prospective study involving 29 patients (20 men, nine women) who underwent surgery using the WALANT technique for lower limb pathologies. The patients were divided into two groups: 10 in the soft tissue surgery group and 19 in the bone surgery group. No significant differences were found in terms of age, sex, or surgical side between the groups. The results demonstrated that the WALANT technique provided adequate pain control and effective hemostasis without the use of a tourniquet in both types of surgery. Three patients in the soft tissue group and one in the bone surgery group required additional solution, and the use of electrocautery was similar in both groups. Most patients reported high satisfaction with the anesthesia and recommended its use, although mild complications, such as numbness and persistent pain, were observed, along with one case of skin necrosis requiring flap surgery. These findings indicate that WALANT is an effective and safe technique for lower limb surgeries, although its proper application is crucial to minimize complications.

Wright et al. [14] conducted a prospective, comparative study with 40 patients undergoing forefoot surgery to evaluate perioperative pain and anxiety with local anesthesia without a tourniquet versus general anesthesia. No significant differences were observed in preoperative pain (*p* = 0.500) or anxiety (*p* = 0.820) between the two groups. However, patients who received local anesthesia without a tourniquet reported significantly lower postoperative pain (*p* < 0.001) and anxiety (*p* < 0.001). During the operation, these patients experienced minimal pain (M = 0.17, SD = 0.32) and anxiety (M = 1.33, SD = 1.74). The results suggest that local anesthesia without a tourniquet is well tolerated and offers significant advantages in terms of reducing postoperative pain and anxiety, improving patient experience, and facilitating faster recovery.

Liszka & Gądek [15] analyzed 80 patients undergoing ankle arthroscopy in a retrospective study. They found that the use of preventive local anesthesia combined with general or spinal anesthesia significantly reduced postoperative pain. These results advocate for a multimodal approach to optimize postoperative pain control.

Ozkan et al. [16] evaluated 105 diabetic patients undergoing distal foot amputation with ultrasound-guided peripheral nerve blocks (PNB). The technique was effective for pain management and showed no complications, highlighting its safety in high-risk populations, including those on anticoagulant therapy.

Luo et al. [17] conducted a prospective comparative study on 20 patients comparing subparaneural and extraparaneural lidocaine injections for popliteal sciatic nerve blocks. Subparaneural injections resulted in faster onset, reduced anesthetic volume, and higher patient satisfaction, indicating greater efficiency and effectiveness.

Iborra et al. [18] studied 61 patients with tarsal tunnel syndrome, using ultrasound-guided lidocaine infiltration (USLIT) as a diagnostic and therapeutic technique. The study reported improvements in symptoms and nerve conduction velocity, positioning USLIT as a useful tool for both diagnosis and treatment.

Bakaes et al. [19] retrospectively reviewed a large cohort undergoing elective foot and ankle surgery with additive regional anesthesia (ARS). While ARS increased operative time, it reduced hospital stays and improved recovery. However, higher complication rates were observed in midfoot and forefoot procedures, suggesting selective use is warranted.

Zhu et al. [20] compared general endotracheal anesthesia (GEA) and PNB in 262 diabetic patients undergoing foot ulcer surgery. PNB provided better hemodynamic stability, lower postoperative pain, and fewer complications. These findings support PNB as a superior option for managing diabetic foot procedures.

These findings highlight the growing body of evidence supporting the use of local and regional anesthesia techniques in podiatric surgery. The effectiveness, safety, and clinical implications of these approaches continue to be evaluated, providing valuable insights for optimizing anesthesia protocols and enhancing patient care outcomes in foot and ankle procedures.

## 4. Discussion

The reviewed studies address the effectiveness and safety of various anesthetic techniques in podiatric procedures. While all studies concur on the efficacy of these techniques, notable differences are observed and analyzed in detail.

Bilgetekin et al. [12] and Çetin et al. [13] focused on the WALANT technique for foot and ankle procedures. Both studies highlighted its effectiveness in controlling pain and anxiety, with high patient satisfaction and rapid hospital recovery. However, Çetin et al. reported minor complications, particularly in bone tissue surgeries, suggesting that the complexity of the procedure may influence the outcomes and safety of the technique. This underscores the importance of proper application and the surgeon’s expertise in minimizing risks.

Wright et al. [14] compared local anesthesia without a tourniquet to general anesthesia, finding that the former provides significant advantages in reducing postoperative pain and anxiety. This difference may be explained by the lower invasiveness of local anesthesia and the absence of side effects associated with general anesthesia. Additionally, keeping the patient awake and communicative during the procedure may contribute to an improved patient experience.

Liszka & Gądek [15] evaluated the combination of preventive local anesthesia with general or spinal anesthesia, highlighting a significant reduction in postoperative pain. The efficacy of this combination can be attributed to the synergy between techniques, where preventive local anesthesia reduces the sensitivity of the surgical area, complementing the effects of general or spinal anesthesia. This suggests that a multimodal approach may be more effective for managing postoperative pain.

Ozkan et al. [16] and Zhu et al. [20] investigated the use of peripheral nerve blocks in diabetic patients. Both studies demonstrated that this technique provides adequate pain control with few complications, emphasizing its relevance in patients with preexisting conditions that increase surgical risks. The hemodynamic stability observed in the study by Zhu et al. suggests that peripheral nerve blocks are a safer option compared to general anesthesia in diabetic patients, who are at greater risk of intraoperative complications.

Luo et al. [17] compared subparaneural and extraparaneural lidocaine injections, demonstrating that the subparaneural injection is more effective for pain control and patient satisfaction. This difference may be due to the closer proximity of the subparaneural injection to the nerve, allowing for more efficient anesthetic distribution and a faster onset of anesthesia.

Iborra et al. [18] and Bakaes et al. [19] evaluated ultrasound-guided techniques, emphasizing the diagnostic and therapeutic precision offered by this technology. Ultrasound guidance improves the accuracy of injections, which could explain the reduction in complications and the improved pain control observed in these studies.

Based on the findings, this review demonstrates that local anesthesia techniques in podiatry are effective and safe. However, the choice of technique depends on multiple factors, including the type of surgery, patient conditions, and surgeon experience. Techniques such as WALANT and ultrasound-guided peripheral nerve blocks stand out for their efficacy and low incidence of complications, but proper application is necessary to maximize their benefits. The differences observed among studies suggest that a personalized approach, considering the specific characteristics of each patient and procedure, could optimize clinical outcomes. Based on the synthesis of current evidence, several recommendations can be made regarding choosing local anesthesia techniques in podiatry. The WALANT technique appears especially advantageous for minor to moderate soft tissue and bone procedures, particularly in outpatient settings, due to its effectiveness, safety, and elimination of tourniquet-related discomfort. However, caution should be exercised in procedures involving complex bone reconstruction or patients with vascular compromise, and alternative or adjunctive techniques may be more appropriate.

Ultrasound-guided peripheral nerve blocks (PNB) are recommended for high-risk patients, such as those with diabetes or receiving anticoagulation therapy, especially in procedures like distal amputations or debridement. These techniques offer excellent hemodynamic stability and pain control with a low complication rate.

For more extensive surgeries, such as midfoot or hindfoot reconstructions, combining preventive local anesthesia with general or spinal anesthesia may enhance postoperative pain management, as supported by the evidence. Nevertheless, additive regional blocks should be used cautiously in midfoot/forefoot surgeries where an increased rate of complications has been observed.

Finally, subparaneural injection techniques are particularly effective for achieving rapid onset and improved analgesia in nerve blocks, although proper training and anatomical knowledge are crucial to minimize potential nerve injury.

These recommendations, derived from the available literature, aim to assist clinicians in selecting the most appropriate anesthetic technique based on patient condition, surgical complexity, and available resources, thus promoting safer and more effective podiatric practice.

### 4.1. Recommendations for Practice and Future Research

For clinical practice in podiatry, the adoption of advanced local anesthesia techniques that have proven effective and safe in recent studies is recommended. In particular, the WALANT (Wide-Awake Local Anesthesia No Tourniquet) technique is a favorable option for foot and ankle procedures. This method allows surgical interventions to be performed without the need for a tourniquet and with the patient awake under local anesthesia. Studies such as those by Bilgetekin et al. [12] and Çetin et al. [13] have documented positive results, including significant reductions in intraoperative pain, lower incidence of postoperative complications, and faster hospital recovery. Therefore, implementing WALANT could enhance the patient experience and optimize clinical outcomes in podiatric practice.

Furthermore, ultrasound-guided peripheral nerve blocks (PNB) are a safe and effective technique for pain management in diabetic foot patients undergoing procedures such as distal amputation or debridement. Studies such as that by Ozkan et al. [16] have emphasized that this anesthetic strategy provides adequate pain control, reduces the need for additional analgesics, and improves intraoperative hemodynamic stability. High patient satisfaction and the absence of severe complications support the recommendation to integrate ultrasound-guided PNB into the anesthetic protocol for complex podiatric surgeries.

In terms of postoperative pain management, the combination of preventive local anesthesia with general or spinal anesthesia emerges as a promising strategy. This approach has demonstrated significant efficacy in reducing pain during the first postoperative hours, minimizing the need for additional analgesics, and improving the overall patient experience. Liszka & Gądek [15] found that this combination optimizes pain management and positively influences postoperative recovery and patient satisfaction. Therefore, this strategy should be considered in anesthetic planning for elective foot and ankle procedures, particularly in patients susceptible to postoperative complications.

As a result, clinical practice in podiatry would benefit significantly from adopting advanced local anesthesia techniques such as WALANT, ultrasound-guided peripheral nerve blocks, and the combination of preventive local anesthesia with other methods. Solid scientific evidence supports these recommendations, emphasizing efficacy, safety, and patient satisfaction. However, continued research and evaluation of these techniques in various clinical contexts are essential to optimize outcomes and establish more specific clinical guidelines in podiatry.

### 4.2. Limitations and Considerations for Implementation

Despite their evident benefits, advanced local anesthesia techniques in podiatry also present limitations that must be considered before widespread implementation in clinical practice.

One major limitation is the learning curve associated with techniques such as WALANT and ultrasound-guided peripheral nerve blocks. These methodologies require specialized technical skills from healthcare professionals to ensure proper administration and minimize the risk of complications. Adequate training and practical experience are essential to guarantee optimal outcomes and patient safety during and after the procedure.

Another factor to consider is the variability in individual patient responses to different anesthetic techniques. Factors such as specific patient anatomy, preexisting medical conditions, and personal pain sensitivity can influence the effectiveness of local anesthesia. This may require adjustments in anesthetic doses or technique selection, highlighting the importance of a thorough and individualized evaluation before the intervention.

Additionally, although the reviewed studies suggest a low incidence of severe complications associated with techniques such as WALANT and ultrasound-guided peripheral nerve blocks, vigilance is crucial for potential adverse effects. Some studies have reported minor complications, such as transient numbness, persistent pain, or rare skin necrosis cases (Çetin et al., [13]). Continuous monitoring and quickly managing complications are essential to minimize risks and ensure patient safety.

Finally, while combining preventive local anesthesia with general or spinal anesthesia has shown significant benefits in reducing postoperative pain, it is essential to consider the possible interaction between different anesthetic agents and medications used during surgery. Coordinated and carefully managing these combinations is crucial to avoid adverse effects and optimize clinical outcomes.

### 4.3. Future Prospects in Local Anesthesia Techniques for Podiatry

Based on current evidence and limitations, several promising directions could further improve the practice of advanced local anesthesia techniques in podiatry.

A key perspective is the continued innovation in technology and surgical techniques. For example, developing advanced imaging systems and robotics could enhance precision and efficacy in local anesthesia administration, particularly in complex or high-precision procedures, such as peripheral nerve surgery. Integrating these technologies could allow for better visualization of relevant anatomical structures and more precise anesthetic administration, reducing risks and improving clinical outcomes.

Additionally, ongoing research in anesthetic pharmacology is crucial. The development of new local anesthetic agents with improved safety profiles and prolonged duration of action could expand the options available to podiatry healthcare professionals. These advances could lead to even more effective and safer anesthetic techniques, enhancing patient experience and reducing postoperative recovery times.

Another promising aspect is the personalization of local anesthesia. As medicine advances toward a more personalized approach, the ability to select anesthetic techniques and dosages based on specific patient characteristics—such as pain response and individual anatomy—could further optimize outcomes. This could include using biomarkers or genetic testing to predict individual responses to different anesthetic agents, enabling more precise and personalized care.

Finally, interdisciplinary collaboration among podiatrists, anesthesiologists, pharmacologists, and medical technology specialists is crucial. Such collaboration can facilitate knowledge exchange, promote joint research, and accelerate the implementation of technological advancements in daily clinical practice.

In summary, the future of advanced local anesthesia techniques in podiatry focuses on technological innovation, developing new anesthetic agents, personalized treatment, continuous education, and interdisciplinary collaboration. These areas offer a promising path toward significant improvements in podiatric procedures’ safety, efficacy, and patient experience, highlighting the potential for positive transformation in future clinical practice.

## 5. Conclusions

Local anesthesia techniques have proven to be effective in reducing pain during podiatric procedures, yielding positive outcomes in terms of pain intensity and patient satisfaction. Their safety profile is high, with a low incidence of severe complications. When comparing different techniques and local anesthetics, local anesthesia without a tourniquet offers significant advantages in reducing postoperative pain and anxiety compared to general anesthesia. Additionally, techniques such as subparaneural injection have been shown to be more effective for pain control and patient satisfaction. Factors such as the proper application of anesthetic techniques, the surgeon’s experience, and the patient’s individual characteristics play a crucial role in determining the efficacy and safety of local anesthesia in podiatry.

## Figures and Tables

**Table 1:** Selected Studies.

Reference	Design	Results	Clinical Implication
Bilgetekin et al. [12]	Retrospective	31 patients (22 men, 9 women; mean age 40 ± 16 years)	The WALANT technique is effective and safe for foot and ankle injuries, with low anxiety and pain levels, and no complications. WALANT can enhance patient experience and optimize hospital resources for foot and ankle injuries.
Çetin et al. [13]	Prospective	29 patients (20 men, 9 women)	Adequate pain control and hemostasis in lower limb surgeries, with high patient satisfaction. WALANT is safe and effective, though large-scale studies are needed for validation.
Wright et al. [14]	Prospective, comparative	40 patients	Lower postoperative pain and anxiety with local anesthesia without a tourniquet compared to general anesthesia. Local anesthesia without a tourniquet can improve patient experience and accelerate forefoot surgery recovery.
Liszka & Gądek [15]	Retrospective	80 patients, assigned to 4 groups	Lower postoperative pain with preventive local anesthesia combined with general or spinal anesthesia. Combining preventive local anesthesia with general or spinal anesthesia can optimize postoperative pain control and improve patient experience.
Ozkan et al. [16]	Retrospective	105 patients with diabetic foot	Peripheral nerve block is effective and safe for distal foot amputation in diabetic patients, with no complications. Ultrasound-guided PNB is suitable for high-risk patients, optimizing postoperative pain control.
Luo et al. [17]	Prospective, comparative	20 patients	Subparaneural injection reduces anesthesia dose and onset time, improving patient satisfaction. Subparaneural injection for the popliteal sciatic nerve block can enhance pain control and patient satisfaction.
Iborra et al. [18]	Retrospective	61 patients (23 men, 38 women; mean age 51 years)	Improvement in symptoms and nerve conduction velocity with ultrasound-guided lidocaine infiltration for tarsal tunnel syndrome. USLIT can confirm tarsal tunnel syndrome diagnosis and predict postoperative improvement, serving as a valuable diagnostic tool.
Bakaes et al. [19]	Retrospective	Large cohort of adult patients from 2006 to 2020	ARS combined with general anesthesia increases operative time but reduces hospital stay in foot and ankle surgeries. ARS may influence hospital management and postoperative outcomes, though it increases complications in midfoot/forefoot surgeries.
Zhu et al. [20]	Retrospective	262 patients (117 with GEA, 145 with PNB)	PNB provides hemodynamic stability and better postoperative analgesia compared to general anesthesia for diabetic foot ulcers. PNB is a safe and effective option for managing diabetic foot ulcers, offering benefits without increasing postoperative risks.

## Data Availability

The raw data supporting the conclusions of this article will be made available by the authors on request.

## Abbreviations

The following abbreviations are used in this manuscript:

ASA	American Society of Anesthesiologists (classification for patient health status)
GEA	General Endotracheal Anesthesia
PNB	Peripheral Nerve Block
USLIT	Ultrasound-Guided Lidocaine Infiltration Technique
WALANT	Wide-Awake Local Anesthesia No Tourniquet
VAS	Visual Analog Scale
